# A muscle synergy-based method to estimate muscle activation patterns of children with cerebral palsy using data collected from typically developing children

**DOI:** 10.1038/s41598-022-07541-5

**Published:** 2022-03-04

**Authors:** Mohammad Fazle Rabbi, Laura E. Diamond, Chris P. Carty, David G. Lloyd, Giorgio Davico, Claudio Pizzolato

**Affiliations:** 1 Griffith Centre of Biomedical and Rehabilitation Engineering (GCORE), Menzies Health Institute Queensland, and Advanced Design and Prototyping Technologies Institute, Griffith University, QLD 4222 Southport, Australia; 2 Department of Orthopaedic Surgery, Children’s Health Queensland Hospital and Health Service, Brisbane, QLD 4101 Australia; 3 Research Development Unit, Caboolture and Kilcoy Hospitals, Metro North Hospital and Health Service, Brisbane, QLD 4101 Australia; 4 Department of Industrial Engineering, Alma Mater Studiorum, University of Bologna, 40136 Bologna, Italy; 5 Medical Technology Lab, IRCCS Istituto Ortopedico Rizzoli, Bologna, Italy

**Keywords:** Computational neuroscience, Biomedical engineering, Neurological disorders

## Abstract

Preparing children with cerebral palsy prior to gait analysis may be a challenging and time-intensive task, especially when large number of sensors are involved. Collecting minimum number of electromyograms (EMG) and yet providing adequate information for clinical assessment might improve clinical workflow. The main goal of this study was to develop a method to estimate activation patterns of lower limb muscles from EMG measured from a small set of muscles in children with cerebral palsy. We developed and implemented a muscle synergy extrapolation method able to estimate the full set of lower limbs muscle activation patterns from only three experimentally measured EMG. Specifically, we extracted a set of hybrid muscle synergies from muscle activation patterns of children with cerebral palsy and their healthy counterparts. Next, those muscle synergies were used to estimate activation patterns of muscles, which were not initially measured in children with cerebral palsy. Two best combinations with three (medial gastrocnemius, semi membranous, and vastus lateralis) and four (lateral gastrocnemius, semi membranous, sartorius, and vastus medialis) experimental EMG were able to estimate the full set of 10 muscle activation patterns with mean (± standard deviation) variance accounted for of 79.93 (± 9.64)% and 79.15 (± 6.40)%, respectively, using only three muscle synergies. In conclusion, muscle activation patterns of unmeasured muscles in children with cerebral palsy can be estimated from EMG measured from three to four muscles using our muscle synergy extrapolation method. In the future, the proposed muscle synergy-based method could be employed in gait clinics to minimise the required preparation time.

## Introduction

The coordinated activation of multiple muscles during rhythmic movements, such as walking, is commonly referred to as a muscle synergy^1^. Muscle synergies reflect motor control arising from the interaction between descending supraspinal motor commands and sensory inflow from peripheral mechanoreceptors^2^, and can be used to gauge motor impairments in people with neurological conditions^3,4^, including stroke survivors^5^, children with cerebral palsy (CP)^6^, and individuals with spinal cord injury^7^ and Parkinson’s disease^8^.

Muscle synergies are extracted from processed electromyograms (EMG), known as muscle activation patterns, using mathematical factorisation methods^9^. These methods assume recorded muscle activation patterns to be linear combinations of excitation primitives, which represent the temporal profile of muscle synergies^10,11^. Linearly combining muscle synergies after extraction produces a synergy-reconstructed muscle activation pattern and a reconstruction error^11^. This error decreases with increasing number of extracted muscle synergies; three to five muscle synergies can typically generate muscle activation patterns that account for variance (VAF) of at least 80% in healthy population and in children with CP^3,6^. The number and location of muscles included in a synergy analysis directly impact the reconstruction error^12^. It is not known which set of muscles generate muscle synergies that best describe how muscles are activated synchronously during walking in children with CP.

Muscle synergies have recently been proposed as a tool to assess the efficacy of neuro-rehabilitation^13,14^. The temporal profile of muscle synergies was altered in children with CP after a botulinum toxin type A injection^14^ and after selective dorsal rhizotomy^15^. The former temporarily inhibits signal from nervous system to block muscle activity^13,14^ and the latter is a single event surgical procedure to decrease spasticity through reduction of the excitatory input of dorsal nerve rootlets^16^. Both treatment groups demonstrated improvements in gait performance, though a direct link with observed synergistic changes was not established^14,17^. Children with CP who have high functional impairment, as per the gross motor function classification scale (GMFCS)^18^, have been shown to use muscle synergies that are fewer than, and a subset of, those observed in their TD counterparts during walking at self-selected speed^3,19^. An increased number of muscle synergies was also associated with functional improvements during walking in children with CP^17,20^. However, studies^14,19,21^ suggested that the *number* of muscle synergies may be difficult to change with an intervention, instead proposing the temporal profile of muscle synergies to be a viable target for treatment of children with CP.

A comprehensive evaluation of muscle coordination in children with CP requires up to 11 EMG electrodes to be placed on a single leg^22^, but usually only four to five EMGs are collected in gait clinics^20^. A synergy extrapolation method^23^ was proposed to reconstruct a set of eight unmeasured muscle activation patterns from 16 experimental EMG recordings of one stroke survivor. Specifically, lower limbs muscle activation patterns recorded from eight muscles of each of the impaired and unimpaired leg were used as input for a synergy extrapolation method to estimate other eight unmeasured muscle activation patterns of the respective legs separately during walking^23^. Although novel, this method required EMG from both impaired and unimpaired muscles, which is not always feasible in children with bilateral CP (i.e., diplegia). However, previous studies^19,21^ demonstrated that the muscle synergies from children with CP are a subset of muscle synergies observed in TD children, making it possible to use muscle synergies from TD children to estimate those in children with CP. Although largely unexplored, this approach would allow muscle synergies to be extracted, and unmeasured muscle activation patterns to be predicted, from recordings of only a few lower limb muscles, which could greatly simplify clinical workflows.

Muscle synergy extrapolation methods to estimate unmeasured EMG could also inform muscle activation patterns used for computational modelling, such as neuromusculoskeletal modelling^23,24^. Neuromusculoskeletal models enable understanding the internal biomechanics of musculoskeletal tissues and are becoming popular tools to create personalised rehabilitation and training interventions^25–27^. Minimising the number of EMG sensors required to attain valuable neuromusculoskeletal estimates (e.g. joint moments and contact forces) would facilitate the adoption of these advanced methods outside the research laboratory. A new method must be able to estimate unmeasured muscle activation pattern(s) while preserving the neural and muscular information of measured muscles and not placing extra burden in the EMG data collection procedure.

The aim of this study was to determine whether activation patterns from 10 lower limb muscles could be accurately reconstructed from a subset of experimental EMG recordings in children with CP. We combined muscle activation patterns from TD children with a reduced set of muscle activation patterns from children with CP. Subsequently, a muscle synergy extrapolation method was developed that used the combined CP and TD datasets to estimate activation patterns of 10 lower limb muscles in children with CP. We hypothesised that EMGs from three or four muscles could be used to estimate all major lower limb muscle activation patterns in children with CP using a muscle synergy extrapolation method. We compared estimation performance as a function of the number of extracted muscle synergies and determined the optimal muscles from which EMG should be recorded.

## Methods

### Participants

Previously collected EMG data^28^ from six children with CP and six age-matched TD control participants was used in the study (Table 1). Inclusion criteria for the CP cohort were age between 6 and 14 years old, diagnosis of CP, and ability to walk independently (i.e., without assistive devices and classified as GMFCS level II and I) on level surfaces for 50 m. Participants were excluded if they had received musculoskeletal surgery (e.g., muscle lengthening) or botulinum injection in the 6 months prior to the testing. The participants with CP were diagnosed with unilateral lesions. The study was approved by the Children’s Health Queensland Hospital and Health Service, and Griffith University Human Research Ethics Committees. Participants’ guardians provided written informed consent prior to data collection. All methods were carried out in accordance with relevant guidelines and regulations of Helsinki.

**Table 1:** Demographics (average ± standard deviation) of participants.

	Cerebral palsy	Typically developing
Sex (male/female)	4/2	2/4
Age (years)	10.75 ± 3.39	10.59 ± 2.89
Mass (kg)	35.70 ± 13.60	37.24 ± 14.97
Height (m)	1.42 ± 0.22	1.42 ± 0.19
CP diagnosis	5 GMFCS I, 1 GMFCS II	–

*GMFCS* gross motor function classification scale.

### Electromyography and data processing

An experienced physiotherapist placed wireless surface EMG bipolar sensors (Aurion, Milan, IT, 1000 Hz) on the affected limb of participants with CP and on the right leg of TD participants, as per SENIAM guidelines^29^: lateral gastrocnemius (LG), medial gastrocnemius (MG), soleus (SOL), tibialis anterior (TA), semi membranous (SM), biceps femoris long (BF), sartorius (SR), vastus lateralis (VL), vastus medialis (VM), rectus femoris (RF). While recording EMG, ground reaction forces were synchronously measured from two ground-mounted force platforms (Advanced Mechanical Technology Inc., Watertown, MA, USA. 1000 Hz) to determine the gait event (i.e., heel strike and toe off) with a threshold value of 20 N. EMG signals were recorded for 10–30 gait cycles (i.e., between consecutive toe-off of the same leg), henceforth referred to as trials, per participant. EMG signals were band-pass filtered at 30–400 Hz (zero lag 4th order Butterworth), full wave rectified, and low-pass filtered at 6 Hz (zero lag 4th order Butterworth). The muscle-specific EMG linear envelopes were normalised to the maximum values identified across the processed trials to ensure that they remained within a range of 0–1^28,30^. Next, each of the linear envelopes was interpolated via cubic splines, and reduced to 100 data points^28,30^, which represented a muscle activation pattern. Data matrices were created where the number of rows was equal to the number of measured muscle activation patterns and the number of columns was equal to the number of data points (i.e., 10-by-100).

**Figure 1:**
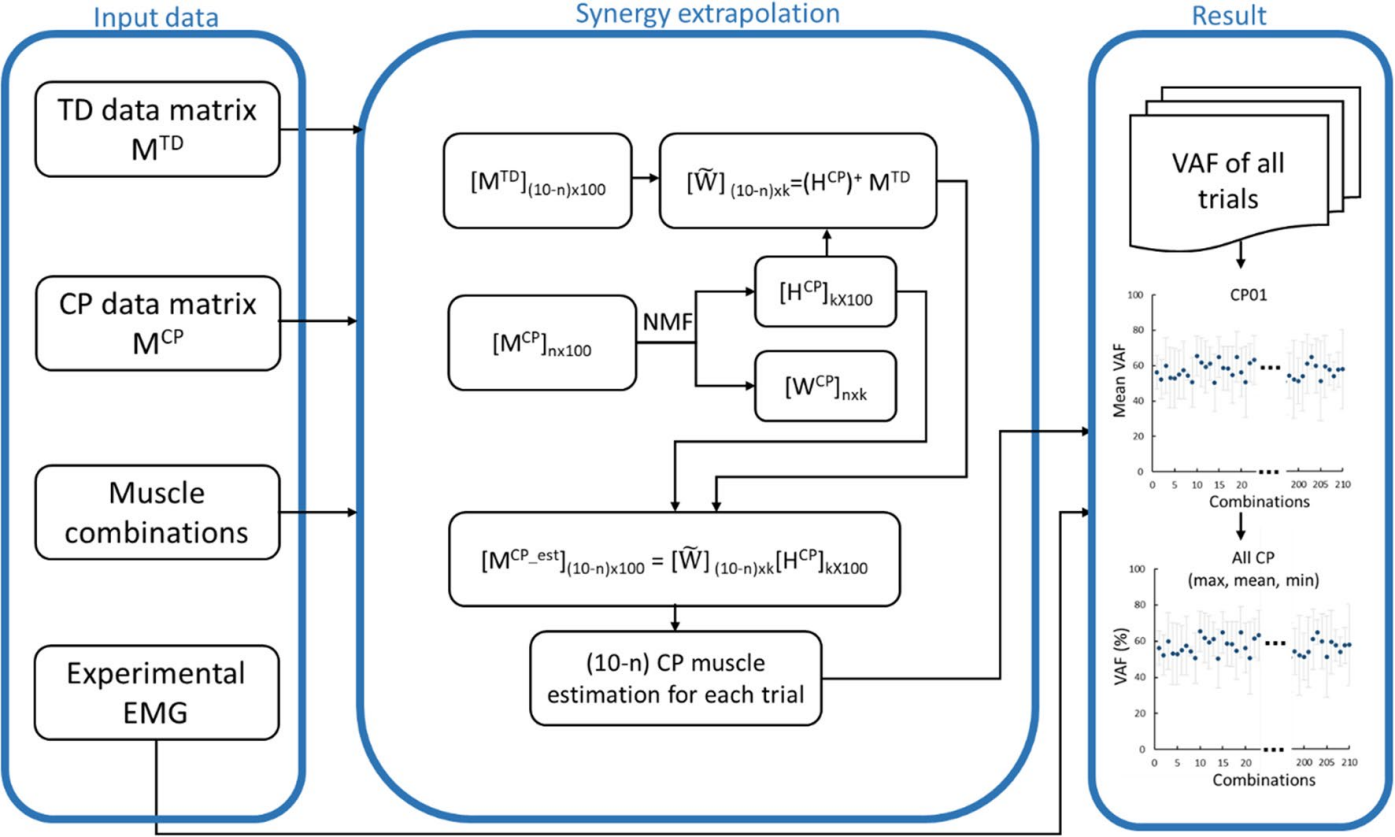
Flowchart of synergy extrapolation method. n, number of muscles included for the participant with CP; (10-n), number of muscles to be estimated in the participant with CP; (.)^+^, Moore–Penrose inverse; H, muscle synergy excitation primitives; k, number of muscle synergies; max, maximum; min, minimum; [${\mathbf{M}}^{\mathrm{CP}}$]_n×100_, CP data matrix; [${\mathbf{M}}^{\mathrm{TD}}$]_(10-n)×100_, TD data matrix; NMF, non-negative matrix factorization; VAF, variance accounted for; W, muscle synergy weights.

For each participant, muscle activation patterns were organised into a 10-by-(100 × p) matrix, where p was the trial number, 10 was the number of measured muscle activation patterns, and 100 was the number of data points. The developed muscle synergy extrapolation process (Fig. 1) was repeated for each trial of each participant with CP, evaluating how all possible combination of measured muscles were able to reconstruct the unmeasured muscles. Critically, only the TD database and the data from the measured muscle of the selected participant with CP were used in the proposed algorithm.

### Muscle combinations

Muscles were grouped into *included* and *excluded* subsets^23^ which corresponded to the measured and unmeasured muscles, respectively. Subsequently, the *included* and *excluded* muscles were selected in CP and TD data matrices, respectively, and used in the synergy extrapolation process (Fig. 1). From a classic data science perspective, the training dataset comprised of the data associated to the *included* muscles of the participant with CP currently being considered combined with the *excluded* muscles from the TD dataset. Conversely, the validation dataset comprised of the data associated to *excluded* muscles of the participant with CP currently being considered. The number of muscle combinations C(n) able to generate an *included* muscle subset of size n was determined as:

1 $$C\left(n\right)=\frac{10!}{n!\left(10-n\right)!}, n=1, \dots , 9$$

where n and (10-n) are the number of *included* and *excluded* muscles, respectively. Muscle activation patterns in the *included* subset were used in synergy extrapolation process while those placed in the estimated subset were omitted from the process. Combinations of three to seven *included* muscles were used to estimate muscle activation patterns of the *excluded* muscles.

### Muscle synergy extrapolation

A muscle synergy extrapolation method was developed to estimate the *excluded* muscle activation patterns by appropriately combining the data matrices from TD participants and selected rows from the data matrix of the participant with CP currently being considered (Fig. 1). For a given combination of *n* muscles in each trial, the CP matrix and TD database matrix were transformed into [${\mathbf{M}}^{\mathrm{CP}}$]_n×100_ and [${\mathbf{M}}^{\mathrm{TD}}$]_(10-n)×100_ matrices respectively, representing the training dataset. Then, CP muscle synergy weights (${\mathbf{W}}^{\mathrm{CP}}$) and excitation primitives (${\mathbf{H}}^{\mathrm{CP}}$) were extracted from the *included* muscle activation patterns of the participant with CP currently being considered ([**M**^CP^]_n﻿×100_) using the non-negative matrix factorization method which was found to be better than other factorisation methods for extracting muscle synergies in walking^9^.

2 $${\mathbf{M}}^{\mathrm{CP}}= {\mathbf{W}}^{\mathrm{CP}}{\mathbf{H}}^{\mathrm{CP}}+{\varvec{e}}$$

where ***e*** represents the reconstruction error.

Next, a set of hybrid muscle synergy weights ($\stackrel{\sim }{{\varvec{W}}}$) were estimated from the *excluded* muscle activation pattern of TD participants ([${\mathbf{M}}^{\mathrm{TD}}$]_(10-n)×100_) using a Moore–Penrose least-squares method, which is a simple and fast algorithm. Additionally, **H**^CP^ and ${\mathbf{M}}^{\mathrm{TD}}$ follow Gaussian distribution (Supplementary Fig. S1) and therefore, the least-square method estimated the best linear unbiased $\stackrel{\sim }{{\varvec{W}}}$ ^31^. The estimated hybrid muscle synergy weights were calculated as:

3 $$\stackrel{\sim }{{\varvec{W}}}=({\mathbf{H}^{\mathrm{CP}})}^{+}\mathbf{M}^{\mathrm{TD}}={\left[{\left({\mathbf{H}}^{\mathrm{CP}}\right)}^{\mathrm{T}}\left({\mathbf{H}}^{\mathrm{CP}}\right)\right]}^{-1}{{\mathbf{H}}^{\mathrm{CP}}{\mathbf{M}}^{\mathrm{TD}}}$$

Finally, muscle activation patterns of remaining (10-n) *excluded* muscles of participants with CP were estimated from the hybrid muscle synergy weights ($\stackrel{\sim }{{\varvec{W}}}$) and excitations primitives (${\mathbf{H}}^{\mathrm{CP}}$) extracted from TD and participants with CP respectively as:

4 $${\mathbf{M}}^{\mathrm{CP}\_\mathrm{est}}=\stackrel{\sim }{{\varvec{W}}}{\mathbf{H}}^{\mathrm{CP}}$$

The estimated muscle activation patterns of *excluded* muscles in participants with CP were then compared to the corresponding experimental muscle activation patterns (${\mathbf{M}}^{\mathrm{Exp}}$), i.e., validation dataset, and used to calculate the total variance accounted for (VAF) as:

5 $$\mathrm{VAF}=1-\frac{\Vert {\mathbf{M}}^{\mathrm{Exp}}-{\mathbf{M}}^{\mathrm{CP}\_\mathrm{est}}\Vert }{\Vert {\mathbf{M}}^{\mathrm{Exp}}\Vert }$$

where $\Vert \Vert$ is the Frobenius norm.

VAF was used as a performance metric to assess how well the muscle activation patterns of the *excluded* muscles were estimated for any given combination of *included* muscles. All possible combinations of *included* muscles were used to calculate the VAF of *excluded* muscles for each trial of each particpant with CP. Next, mean VAF across all trials in each participant with CP was calculated. The best *included* muscle combination was selected based on the highest mean VAF, representing best esimtation accuracy across all six participants with CP. Further we calculated determination of correlation (R^2^) and root mean squared error (RMSE) between the experimental and estimated muscle activation patterns to quantify the estimation errors. In addition to the VAF, R^2^, and RMSE, we introduced another evaluation metric known as Kolmogorov–Smirnov (KS) test^9^ to gauge the neural information preserved in the estimated muscles. Using the KS test, the probability density function (PDF) of the measured and estimated muscle activation patterns in *excluded* set were compared to assess similarity in information content^9^. The KS test results in the occurrence of agreement and maximum dissimilarity metrics, which were used together with the VAF to determine the best combination of *included* muscles.

### Ethics approval and consent to participate

The study was approved by the Children’s Health Queensland Hospital and Health Service, and Griffith University Human Research Ethics Committees. Participants’ guardians provided written informed consent prior to data collection.

### Statistical analyses

Data were checked for normality using Shapiro–Wilk Tests. If data distributions were normal, all performance metrics (i.e., VAF, occurrence of agreement and maximum dissimilarity) across all muscle combinations were compared using a repeated measure analysis of variance (ANOVA) with Bonferroni correction. Otherwise, equivalent non-parametric tests (i.e., Friedman test followed by Wilcoxon signed rank tests) were used. Since this was a feasibility assessment of a novel method, an a priori power analysis could not be conducted to determine sample size. However, a post-hoc power analysis was conducted to determine the statistical power of the comparisons between muscle combinations. IBM SPSS Statistics v26^32^ and GPower v3.1.9 software^33^ were used for ANOVA and post-hoc power analyses, respectively.

## Results

**Figure 2:**
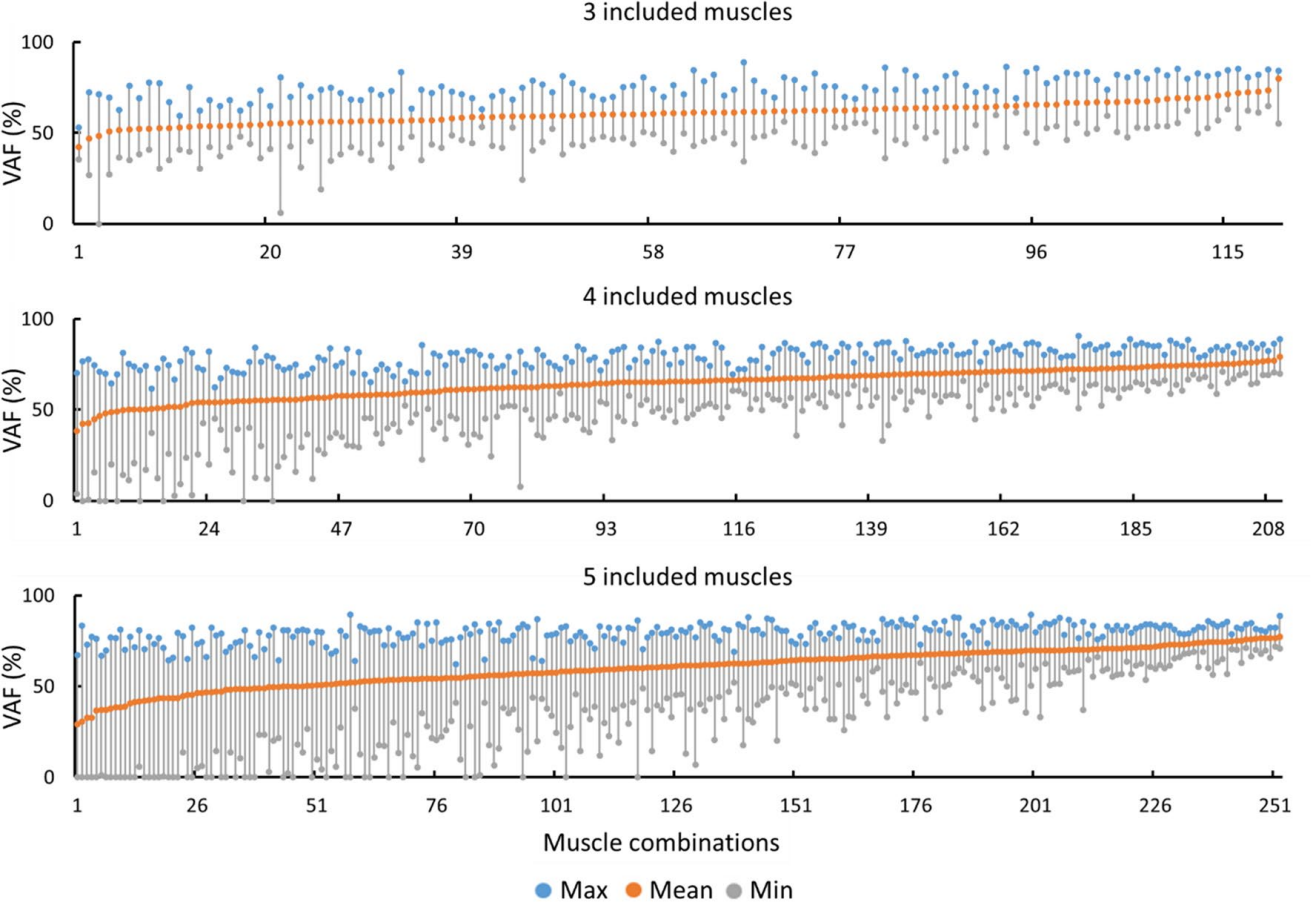
Maximum (max), mean, and minimum (min) variance accounted for (VAF) (%) across all participants with cerebral palsy with three (top), four (middle), and five (bottom) *included* muscles when three muscle synergies were extracted. Results were ranked by mean VAF values to facilitate visualisation.

**Figure 3:**
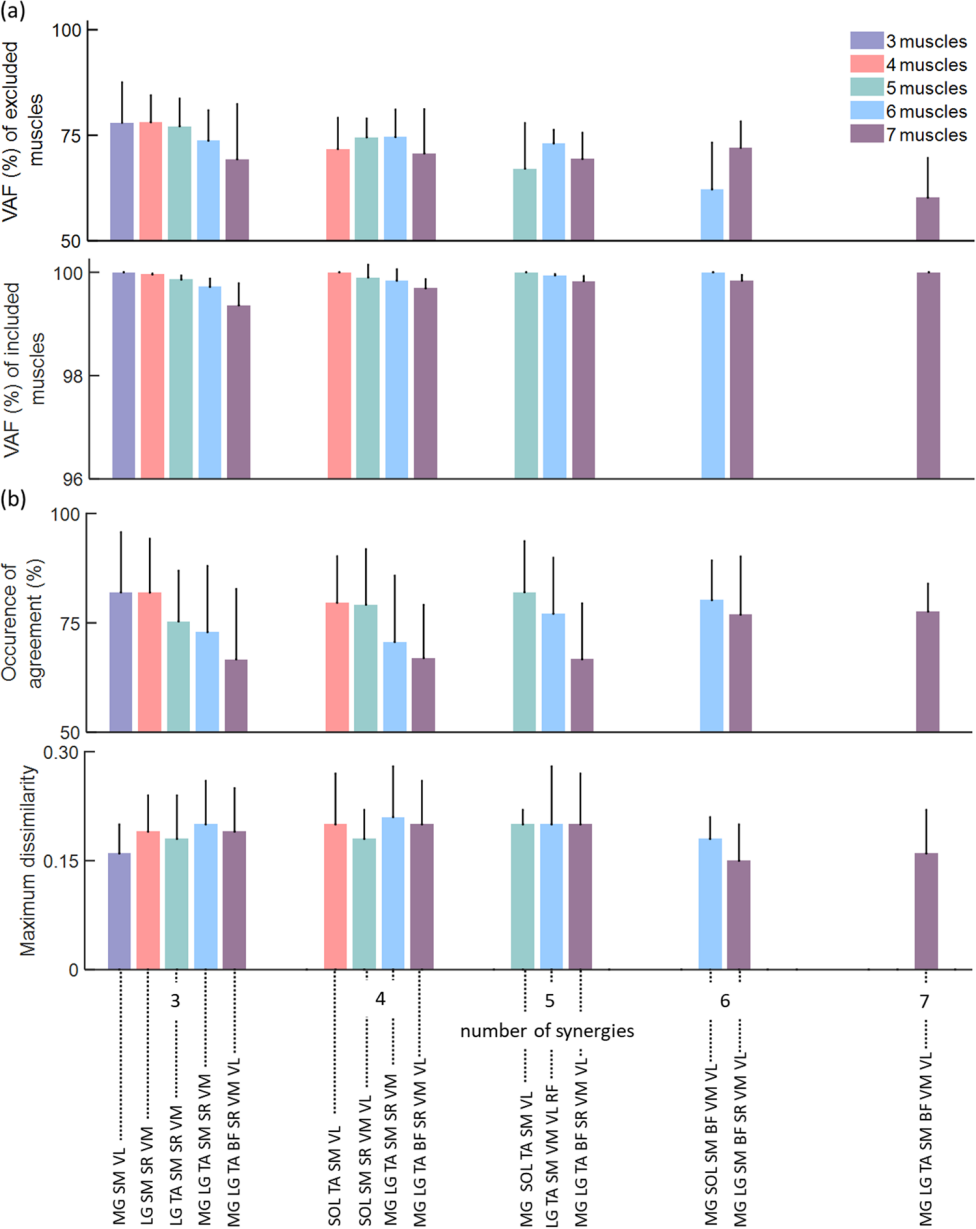
(**a**) Best average VAF across all participants with CP (n = 6) for three to seven *included* muscles when three to seven muscle synergies were extracted. (**b**) KS test results including occurrence of agreement of probability density function between original and estimated muscle activation patterns. Error bars represent the standard deviation across six participants with CP. *VAF* variance accounted for, *BF* Biceps femoris long, *LG* lateral gastrocnemius, *MG* medial gastrocnemius, *RF* rectus femoris, *SR* Sartorius, *SM* semi membranous, *SOL* soleus, *TA* tibialis anterior, *VL* vastus lateralis, *VM* vastus medialis.

Maximum, mean, and minimum VAF (%) across all children with CP were calculated for each combination of *included* muscles from which EMGs needs to be recorded (Fig. 2). The combinations of *included* muscles yielding the best reconstruction of *excluded* muscles, based on mean VAF, were then further analysed to ascertain the best *included* muscle combinations (Table 2, Fig. 3). A post-hoc power analysis revealed that for the given sample size (n = 6) and α = 0.05, the power of comparing VAF values between three and seven, and between four and seven muscles ranged from 0.92 to 0.98, respectively. The effect size was calculated based on the average VAFs across three to seven combinations in each group. At least one of each *included* muscle combinations (Table 2) could estimate the *excluded* muscles with more than 75% VAF when three muscle synergies were extracted. When four muscle synergies were extracted, the highest VAF (74.58%) to estimate four *excluded* muscles was found using a combination of six *included* muscles (MG, LG, TA, SM, SR and VM). Adding more than four muscles in the *included* set did not significantly improve the VAF (p > 0.05). VAFs to estimate the excluded muscles for three to seven included muscles are available in supplementary Table T1. Finally, when using three or more muscle synergies, the VAF for reconstructing the *included* muscles was consistently higher than 99% for all combinations (Fig. 3a). R^2^ and RMSE also resulted in the muscle combinations similar to when using average VAF (Supplementary Table T1).

**Table 2:** Three best *included* muscle combinations when extracting three and four muscle synergies from three and four muscles.

#Muscle	#Synergy	Muscle combination				Mean ± STD VAF (%)
3	3	MG	SM	VL		79.93 ± 9.64
		SOL	SM	VL		73.58 ± 8.40
		MG	TA	VL		72.79 ± 6.61
4	3	LG	SM	SR	VM	79.15 ± 6.40
		SOL	SM	VM	VL	77.18 ± 5.36
		SOL	TA	SM	VM	76.94 ± 4.82
	4	SOL	TA	SM	VL	71.74 ± 7.40
		MG	TA	SM	VL	71.06 ± 7.55
		MG	SOL	SM	VL	70.45 ± 11.94

*LG* lateral gastrocnemius, *MG* medial gastrocnemius, *SR* Sartorius, *SM* semi membranous, *SOL* soleus, *STD* standard deviation, *TA* tibialis anterior, *VAF* variance accounted for, *VL* vastus lateralis, *VM* vastus medialis.

**Figure 4:**
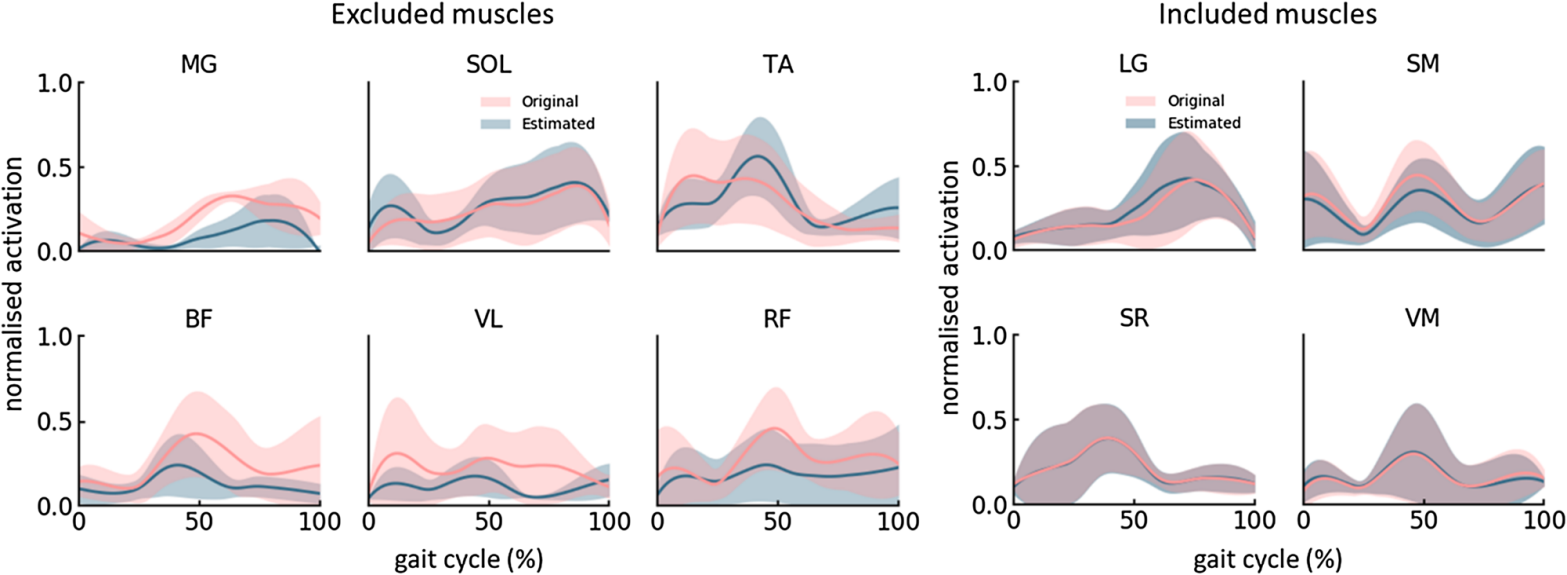
Original and estimated muscle activation patterns of six *excluded* and four *included* muscles when three muscle synergies were extracted. The shaded regions represent the standard deviation across six participants with CP. *BF* Biceps femoris long, *LG* lateral gastrocnemius, *MG* medial gastrocnemius, *RF* rectus femoris, *SR* Sartorius, *SM* semi membranous, *SOL* soleus, *TA* tibialis anterior, *VL* vastus lateralis, *VM* vastus medialis.

The occurrence of agreement, which is the first of the two output of the KS test, between PDF of original and estimated muscle activation patterns decreased when the number of muscles in the *included* group increased (Fig. 3b). Also, extraction of more than three muscle synergies did not significantly improve the occurrence of agreement when four or more *included* muscles were used. The maximum dissimilarity, which is the second output of the KS test, between PDF of original and estimated muscle activation patterns were not significantly different across varying number of *included* muscles and synergies (p > 0.05). An example of estimated muscle activation patterns of six *excluded* muscles using four *included* muscles is presented in Fig. 4.

## Discussion

This study aimed to identify a minimal set of muscles from which EMG could be measured to estimate the muscle activation patterns of all major lower limb muscles in children with CP during walking. We showed that 10 muscle activation patterns of children with CP could be reconstructed using experimental EMG data from three and four muscles with > 70% VAF. A muscle synergy extrapolation method was developed to estimate unmeasured (i.e., *excluded*) muscles of children with CP by only using a minimal number of muscle recordings (i.e., *included*) and a database of muscle activation patterns from TD participants. Using our method, the number of EMG recording sites could be reduced to only three or four, which might lead to a reduction of the subject’s preparation time prior to clinical gait analysis. Critically, our method could also be extended to other neurologically impaired populations (e.g., individuals with stroke and spinal cord injury).

All possible muscle combinations from 10 muscles were examined to select the best combination of *included* muscles (Fig. 2). Although generated by an algorithm which has no connection with biomechanical function, the optimal combination of muscles from which EMG should be measured included biarticular actuators across hip, knee, and ankle joints. At least one muscle from the calves (MG), hamstrings (SM) and quadriceps (VL) muscle groups should be *included* when choosing a 3-muscle combination (Table 2). However, if some of the *included* muscles could not be measured (e.g., excessive atrophy, difficult placement of EMG electrodes, etc.), they could be replaced by other muscles based on the second or third best average VAF across all participants with CP (Table 2, Supplementary Table T1). For example, TA was found in a top three combination when both three and four muscles were included, and both three and four muscle synergies were extracted. Therefore, TA could be a desirable candidate for recording in clinical assessments of children with CP. Identified best muscle combinations using average VAF were also similar when using R^2^ and RMSE (Supplementary Table T1). While our results were determined from a small cohort of children with CP, all the best-ranked muscle combinations had muscles spanning all three joints strongly aligned with the biomechanics of walking, possibly suggesting that our method could provide similar results when assessed on a larger number of participants.

We opted to select muscle combinations based on the best average VAF across all participants with CP. This reflects the idea that a combination of muscles ensuring a good reconstruction for all participants is preferable to a combination of muscles that is excellent for only some of the participants. For example, the maximum VAF for a given muscle combination and a participant might be over 90% (Fig. 3) which might not be true for another participant with the same muscle combination. In a previous study^23^, the best *included* muscle combination was selected based on two criteria: (1) VAF cut-off value (90%), and (2) most frequent muscles which appear in the top 10% VAFs among all muscle combinations. However, a 90% VAF threshold could eliminate all muscle combinations for some of our participants. Muscles that appear most frequently in the *included* muscle combinations and that satisfy the 90% VAF threshold are also likely to vary across participants. Using this approach will make it impossible to identify the best combination of muscles for each patient in a clinical setting without a complete dataset of recorded EMG, so one might as well use the recorded EMG and not resort to EMG reconstruction. In addition to the reconstruction VAF, it is also necessary to verify if information conveyed by the original muscle activation patterns is preserved by estimated muscle activation patterns adequately.

Previous studies^3,17,34^ that analysed EMG data of a large number of participants with CP, only recorded data from only five lower limb muscles during gait analysis. These studies were conducted in clinics over a period spanning 10–15 years, and it is unclear whether it would have been possible to include a larger number of EMG recordings without disrupting the clinical workflow. However, it is plausible to assume that the choice of acquiring a low number of employed EMG sensors might be a matter of practicality, cost, and patient’s comfort. Our method enables sophisticated analysis in clinical environment while maintaining an agile workflow. Specifically, a complete set of muscle activation patterns could be extracted from these datasets to inform neuromusculoskeletal modelling paradigms able to calculate internal biomechanics (e.g., muscle forces and joint contact forces)^28,35^, which are promising clinical targets for conservative and surgical interventions^36^.

Previous studies^17,19,37,38^ showed that individuals with CP were likely to employ simplified motor control strategies compared with TD individuals, suggesting an inverse relationship between increasing level of motor impairment, as classified by GMFCS, and number of muscle synergies. Specifically, for a given number of muscle synergies, EMG from individuals with severe impairment could be reconstructed with a larger VAF than in individuals with less severe impairments^3,20^. In this study we found the reconstruction VAF in participants whose movement functions were classified as GMFCS I (n = 5) was 4.3(± 1.9)% lower than that in participants with GMFCS II (n = 1) for any given number of synergies between three to seven. Although our sample size was small, our results are consistent with the findings from previous studies.

Since PDF of EMG data has a direct relationship with peripheral and neural information conveyed by the signal during any muscle activity^39,40^, KS test was used to compare the PDFs of both original and estimated muscle activation patterns^9^. Occurrence of agreement of the PDFs was more than 80% for three, four, and five *included* muscle combinations when three, four, and five muscle synergies were extracted, respectively (Fig. 3b). Additionally, muscle groups of six and seven *included* muscles could estimate the rest of the muscle activation patterns with more than 75% occurrence of agreement when six or more muscle synergies were extracted. The maximum dissimilarity of PDF between the original and estimated muscle activation patterns ranged 0.15–0.2 across all *included* muscle combinations and muscle synergies (Fig. 3b). Overall, the KS test results followed a similar trend to VAF results; however, deterioration of estimation performance was more apparent in the KS test result (Fig. 3b) when more muscles were added in the *included* muscle combinations. For example, extracting three synergies from three and four muscle combinations produced better VAF and KS test results than those results when more synergies were extracted from larger groups of muscles (Table 2). The reason might be the simplified motor complexity in children with cerebral palsy that requires fewer lower limb muscles to coordinate during a walking^3^. Therefore, information was best preserved in the *excluded* muscles using a small number of *included* muscles when fewer muscle synergies were extracted.

This study has some limitations. We assessed all combinations of *included* muscles within a set of EMG collected from 10 major lower limb muscles from a single leg in six participants with CP. To generalise results, future studies should aim to reproduce our findings using a larger cohort. Large databases^3,34^ of EMG from children with CP do exist; however, limited muscles sites are available. Therefore, these databases could not be used to further validate our proposed method. Although the proposed method requires EMG data from TD children measured beforehand, the EMG data only needs to be available in a database and does not need to be collected on sites. Furthermore, it remains unclear whether the proposed muscle synergy extrapolation method based on non-negative matrix factorisation is sufficiently sensitive to capture spasticity provoked EMG activations commonly used to inform clinical decision making in children with CP^41^. Although recent study^42^ found synergy extrapolation method promising, it is yet to explore how neuromusculoskeletal model would perform when synergies extracted from two groups of population (healthy and impaired) are combined and used as input. Future use of the proposed synergy extrapolation method could be combined with neuromusculoskeletal models to predict internal biomechanics in children with CP. However, it remains unclear, and warrants further study, whether the fidelity of reconstructed muscle activation patterns would be sufficient to obtain modelled estimates with comparable accuracy to standard EMG-informed approaches.

## Conclusions

Our muscle synergy extrapolation method accurately estimated muscle activation patterns of unmeasured muscles in individuals with CP when EMG data from only few muscles in children with CP and other muscles in TD children were available. At least one muscle from the quadriceps, hamstring and calf groups should be experimentally measured to obtain adequate estimation of unmeasured lower limbs muscles. Although evaluated by estimating activation patterns of unmeasured muscles in children with CP, the proposed muscle synergy extrapolation method could strongly support applications where collecting large volume of EMG recording is challenging. More importantly our proposed method indicates that creating a substantially large database of the EMGs collected from typically developing children (which is easier to do) would facilitate estimation of muscle activation patterns of children with CP on demand in clinical settings.

## Supplementary Information

Supplementary Information.

## Data Availability

The datasets used in the current study are available from the corresponding author on reasonable request.
