# Supplementary material for: A muscle synergy-based method to estimate muscle activation patterns of children with cerebral palsy using data collected from typically developing children
Source: Sci Rep. 2022 Mar 4;12:3599. doi: 10.1038/s41598-022-07541-5 (PMC8897462; doi:10.1038/s41598-022-07541-5)
Supplement: Supplementary file 1 — Supplementary Information. [file 41598_2022_7541_MOESM1_ESM.docx]

Supplementary material


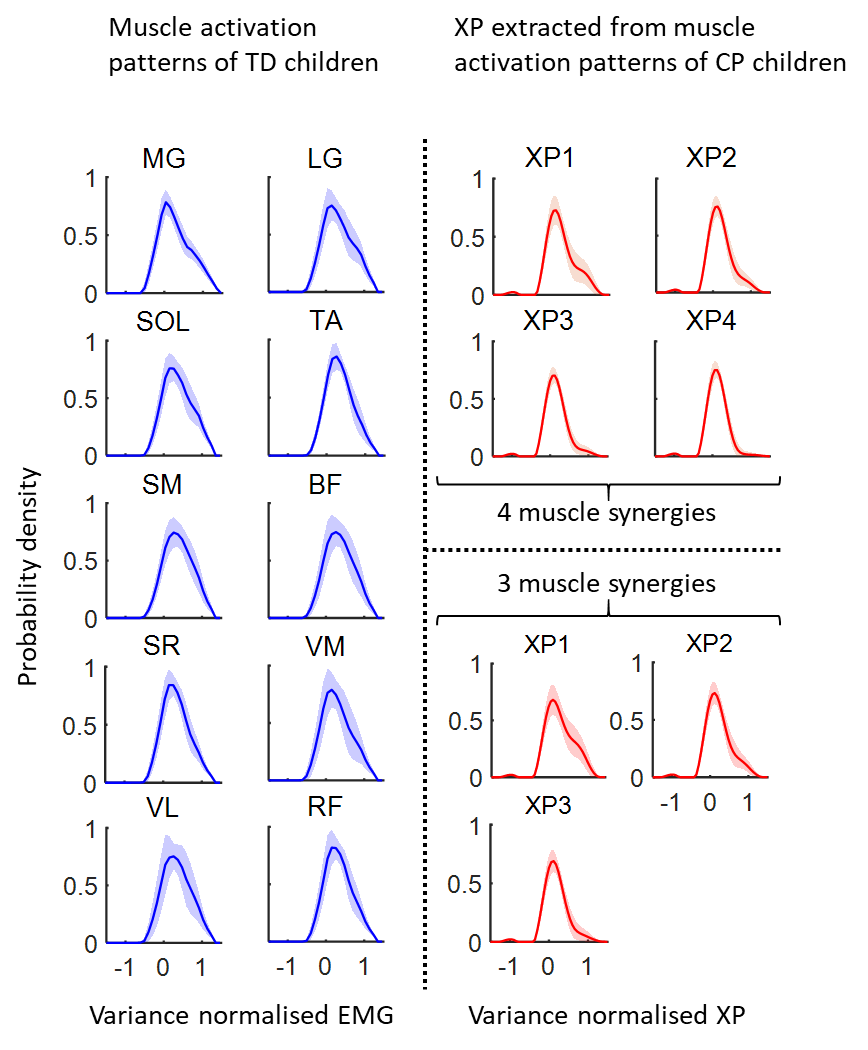


Supplementary Figure S1. Probability density function of muscle activation patterns of TD participants and NMF generated excitation primitives of participants with CP when three and four muscle synergies were extracted. Shaded regions represent the standard deviation across participants.

Supplementary Table T1: best *included* muscle combinations and accuracy in terms of VAF, R^2^ and RMSE for estimating three to seven *excluded* muscles when extracting three to seven muscle synergies.

| #*included* muscle | #synergy | best *included* muscle combinations | mean ±std VAF (%) | mean ±std R^2^ | mean ±std RMSE |
| --- | --- | --- | --- | --- | --- |
| 3 | 3 | MG SM VL | 79.93 ±9.64 | 0.77±0.02 | 0.01±0.01 |
|  |  | SOL SM VL | 73.58 ±8.40 | 0.72±0.03 | 0.01±0.01 |
|  |  | MG TA VL | 72.79 ±6.61 | 0.69±0.04 | 0.01±0.01 |
| 4 | 3 | LG SM SR VM | 79.15 ±6.40 | 0.77±0.02 | 0.01±0.01 |
|  |  | SOL SM VM VL | 77.18 ±5.36 | 0.74±0.01 | 0.02±0.01 |
|  |  | SOL TA SM VM | 76.94 ±4.82 | 0.72±0.03 | 0.03±0.01 |
|  | 4 | SOL TA SM VL | 71.74 ±7.40 | 0.70±0.02 | 0.01±0.01 |
|  |  | MG TA SM VL | 71.06 ±7.55 | 0.69±0.01 | 0.03±0.01 |
|  |  | MG SOL SM VL | 70.45 ±11.94 | 0.67±0.03 | 0.03±0.02 |
| 5 | 3 | LG TA SM SR VM | 77.12 ±6.6 | 0.74±0.01 | 0.01±0.01 |
|  |  | SOL TA SM VM VL | 76.72 ±3.27 | 0.73±0.02 | 0.01±0.01 |
|  |  | LG SOL SM SR VM | 76.49 ±5.82 | 0.73±0.04 | 0.01±0.01 |
|  | 4 | SOL SM SR VM VL | 74.44 ±4.5 | 0.73±0.02 | 0.01±0.01 |
|  |  | MG LG TA SM VL | 74.42 ±4.47 | 0.74±0.04 | 0.02±0.01 |
|  |  | LG SM BF VM VL | 74.12 ±5.67 | 0.71±0.01 | 0.01±0.02 |
|  | 5 | MG SOL TA SM VL | 67.00 ±10.94 | 0.66±0.04 | 0.02±0.01 |
|  |  | MG TA SM VM VL | 66.96 ±9.47 | 0.65±0.02 | 0.02±0.01 |
|  |  | MG SOL TA BF VL | 65.71±12.00 | 0.62±0.01 | 0.01±0.02 |
| 6 | 3 | MG LG TA SM SR VM | 73.75 ±7.28 | 0.72±0.06 | 0.01±0.01 |
|  |  | LG SOL TA SM SR VM | 73.61 ±6.27 | 0.71±0.03 | 0.01±0.01 |
|  |  | LG SOL TA SM VM VL | 73.22 ±5.45 | 0.70±0.02 | 0.03±0.01 |
|  | 4 | MG LG TA SM SR VM | 74.58 ±6.57 | 0.73±0.02 | 0.03±0.01 |
|  |  | MG LG TA SM VM VL | 73.53 ±4.75 | 0.72±0.02 | 0.04±0.01 |
|  |  | MG LG TA BF VM VL | 73.34 ±8.41 | 0.72±0.01 | 0.03±0.02 |
|  | 5 | MG LG TA SM SR VM | 73.77 ±4.40 | 0.72±0.03 | 0.03±0.02 |
|  |  | LG TA SM VM VL RF | 73.06 ±3.20 | 0.71±0.04 | 0.03±0.01 |
|  |  | MG LG TA BF SR VM | 72.91 ±6.33 | 0.70±0.01 | 0.04±0.01 |
|  | 6 | MG TA SM VM VL RF | 62.86 ±10.30 | 0.60±0.02 | 0.03±0.01 |
|  |  | MG SOL TA SM VM VL | 62.65 ±9.04 | 0.61±0.02 | 0.04±0.01 |
|  |  | MG SOL SM BF VM VL | 62.17 ±11.08 | 0.61±0.01 | 0.04±0.02 |
| 7 | 3 | MG LG TA BF SR VM VL | 69.31 ±13.3 | 0.66±0.06 | 0.04±0.01 |
|  |  | LG SOL TA SM SR VM VL | 68.20 ±7.71 | 0.65±0.04 | 0.05±0.03 |
|  |  | MG LG TA SM SR VM VL | 67.18 ±10.16 | 0.62±0.01 | 0.04±0.02 |
|  | 4 | MG LG TA BF SR VM VL | 70.67 ±10.55 | 0.70±0.06 | 0.01±0.01 |
|  |  | MG LG TA SM SR VM VL | 67.40 ±7.17 | 0.64±0.03 | 0.03±0.01 |
|  |  | MG LG SOL SM SR VM VL | 67.03 ±4.08 | 0.64±0.02 | 0.04±0.01 |
|  | 5 | MG LG TA BF VM VL RF | 69.35 ±6.03 | 0.66±0.06 | 0.01±0.01 |
|  |  | LG TA SM BF VM VL RF | 68.44 ±6.78 | 0.65±0.04 | 0.02±0.01 |
|  |  | MG LG TA SM BF VM VL | 66.65 ±8.96 | 0.65±0.01 | 0.02±0.02 |
|  | 6 | MG LG SM BF SR VM VL | 71.99 ±6.3 | 0.69±0.01 | 0.03±0.01 |
|  |  | MG LG TA BF SR VM VL | 69.56±5.86 | 0.68±0.02 | 0.03±0.03 |
|  |  | MG SOL SM BF SR VM VL | 68.68 ±4.39 | 0.66±0.04 | 0.04±0.03 |
|  | 7 | MG LG TA SM BF VM VL | 60.25 ±9.49 | 0.60±0.02 | 0.02±0.01 |
|  |  | MG LG TA SM VM VL RF | 60.18 ±12.12 | 0.60±0.04 | 0.03±0.02 |
|  |  | MG LG TA SM SR VM RF | 59.46 ±10.78 | 0.58±0.04 | 0.04±0.01 |

VAF – variance accounted for; R^2^ –determination of correlation; RMSE – root means squared error;
